# The impacts of COVID-19 on cattle traders and their response in agro-pastoral and pastoral regions in Uganda: A case of Karamoja and Teso cattle traders

**DOI:** 10.1186/s13570-022-00230-y

**Published:** 2022-04-14

**Authors:** John Ilukor, Akello Joyce, Simon Peter Okiror

**Affiliations:** 1 Development Data Group - Survey Unit, World Bank, Kampala, Uganda; 2 School of Agricultural Sciences, Makerere University, Kampala, Uganda

**Keywords:** Agro-pastoral, Pastoral, Cattle traders, COVID-19, Karamoja, Teso, Uganda

## Abstract

The study assessed the economic impact of COVID-19 on cattle traders in the Karamoja and Teso pastoral and agro-pastoral areas in Uganda and their response after the COVID-19 lockdown in 2020. The results reveal that cattle traders were negatively affected by COVID-19 in many ways including reduction in cattle sales, erosion in operating capital, and failure to sell animals while others have diversified or moved to other businesses. Twenty-five per cent of the cattle traders did not sell any animal during the lockdown. A majority of these were from Karamoja (43%) compared to those in Teso sub-region. The decline in cattle sales was significantly higher in Karamoja than in Teso sub-region. However, their recovery was significantly higher in Karamoja than in Teso sub-region because traders in Teso greatly diversified to other economic activities compared to traders in Karamoja sub-region. The traders who lost capital were mainly in Teso sub-region (63%). As expected, there was a sharp decline in the number of cattle buyers from markets outside the study area, mainly from Juba, Kampala, Busia, and Kenya. Coping strategies by cattle traders included crop cultivation (80%), burning charcoal (15%), selling food items (8%), and boda-boda riding (12%), while others did not engage in any economic activity (25%). To mitigate against the pandemic, traders were observing some of the standard operating procedures (SOPs) such as wearing face masks (76.1%), handwashing (19.3%), sanitising (2.3%), and social distancing (2.3%). Traders from Karamoja performed poorly in both diversification and mitigation measures. Based on our findings, recommendations to mitigate the impact of COVID-19 on cattle traders include offering loans to cattle traders through their Village Savings and Loan Association (VSLA), reducing transaction costs, offering mobile phones especially for Karamoja traders, and promoting the adoption of enforcing SOPs to reduce the need for lockdowns and cattle market closures which are detrimental to pastoral livelihood.

## Background

Livestock contributes to people’s livelihoods through numerous channels such as income, food, employment, draft power, hauling services, manure, social status, savings, and insurance, among others (Nyariki and Amwata 2019). For livestock keepers including pastoralists who rely mainly on livestock rearing with mobile herds (Griffith et al. 2020), and agro-pastoralists (crop and livestock farmers) whose livelihood strategy is dependent on keeping livestock, in East Africa  access to livestock markets is an essential component of their livelihood (Aklilu and Catley 2010; Lynch 2020; Roba et al. 2017). Livestock traders play a significant role in the livestock value chains by linking producers, processors, and consumers, thus acting as lifeblood for pastoralists whose areas are remote, less accessible, and sometimes insecure due to cattle raids (Aklilu 2017; Roba et al. 2019; Roba et al. 2017). They streamline the flow of livestock from remote and insecure areas and connect rural or pastoral communities to national and international markets, thus reducing transaction costs and risks related to livestock trade. In addition, they take advantage of the price variation by transacting in spatially and temporally separated markets amidst high transportation costs, insecurity, and unpredictable prices (Aklilu 2017; Little et al. 2014; Roba et al. 2017).

**Fig. 1:**
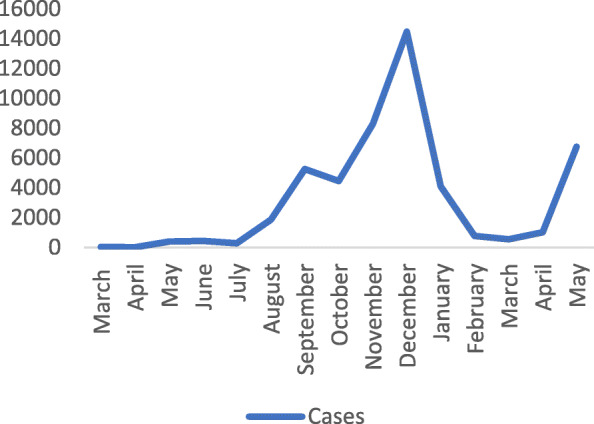
Number of COVID-19 cases from March 2020 to May 2021 as reported by the Ministry of Health in Uganda

**Fig. 2:**
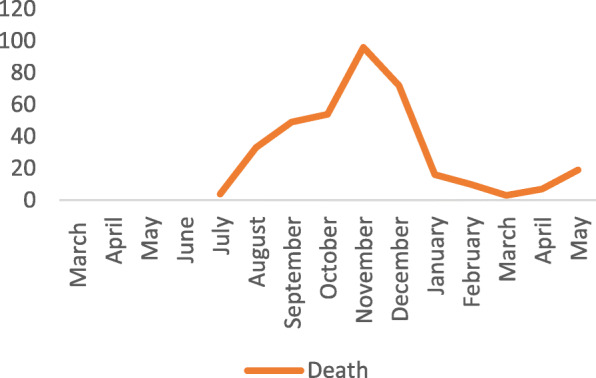
Number of COVID-19 death from March 2020 to May 2021 as reported by the Ministry of Health in Uganda

However, the recent outbreak of the COVID-19 pandemic that disrupted the economic activities across the world, leading to uncertainty and damage to the medium-term economic prospects, has also greatly affected livestock market systems largely arising from attempts to control the spread of the pandemic (Lynch 2020). The pandemic has led so far to the drastic loss of over three million people globally and challenged public health, food systems, and economic activity. In Uganda, the country has registered over 48,000 cases, more than 360 deaths, over 15,147 recoveries and many more still hospitalised between March 2021 and May 2021 with a high number of cases and death registered in November 2020 (Figs. 1 and 2).

In addition to the health impacts, the COVID-19 pandemic has inflicted significant social and economic costs to the economy resulting largely from government measures aimed at slowing COVID-19 transmission. For example, before the first case of the pandemic was registered in Uganda on 21 March 2020, the Government of Uganda had undertaken several actions starting on March 18, including travel restrictions, a 14-day quarantine for all international arrivals, and cancellation of all international conferences and public gatherings, including, but not limited to, religious services, weddings, and concerts. On March 30, the president declared a nationwide curfew from 7 pm to 6:30 am, banned public transportation, and instituted strict regulations for the movement of government and private vehicles as well as the closure of markets (Museveni 2020a, 2020b).

These measures have disproportionately impacted individuals or households (Bell et al. 2020). For example, many people in Uganda who rely on daily wages were unable to go out and work, and many business owners saw their supply chains disrupted and demand drying up (UNICEF 2020). The results from the World Bank High-Frequency Survey conducted in June 2020 revealed that most work stoppages were happening in the non-agricultural sectors largely because of the closure of businesses (World Bank 2020). The main affected individuals were those working in the service sector, transport, and commerce (i.e. buying and selling) while agriculture was the least impacted sector probably because the markets and shops for agricultural inputs, veterinary drugs, and foodstuffs were left open during the lockdown (Lynch 2020). However, the results from the recent rounds of the high-frequency phone survey show that employment rates returned to the pre-lockdown level of 86% with work stoppages occurring largely in the agriculture sector which is probably related to seasonal changes in the labour market (Aziz et al. 2021).

While agriculture was the least affected sector by COVID-19 (FAO 2020; OECD 2020), the sudden closure of livestock markets from March to October 2020 left cattle traders, who are the critical part of the beef value chains with market-ready animals and unable to access buyers (Lynch 2020). The closure of hotels, restaurants, and bars also resulted in reduced demand for cattle and meat/beef which affected the ability of individuals or households that rely on livestock keeping as a livelihood strategy to earn income (Lynch 2020). In the Karamoja of Uganda, government efforts to control the spread of COVID-19 reduced household purchasing power because of loss of income arising from increases in food prices (116% in some areas) beyond the seasonal price increases. The increases in prices were largely driven by the increased cost of local public transport and reduced supply because of market closures. Also, taking account of the seasonal changes, a net decrease in livestock prices of 32% was registered, livestock mortality increased due to limited access to veterinary drugs and services, and losses of livestock increased  due to the resurgence of livestock raids or theft (Catley 2020).

Although the lockdown on cattle markets was lifted on October 8, 2020, there is limited knowledge on how COVID-19 has affected cattle traders in terms of their cattle sales, working capital, and if the traders were able to get back to business. Moreover, although the government has promised to support people or businesses that were affected by COVID-19 as part of the COVID-19 response (URSB 2021), cattle trade or businesses are not part of the target group.

In this study, we assess the economic impact of COVID-19 on cattle traders and their response with the objective of generating possible interventions to the economic recovery of cattle traders (sheep and goat are not included although some trades could be dealing in goats and sheep as well) in Teso and Karamoja sub-regions in Uganda. More specifically, we characterise cattle traders based on the COVID-19 impacts and assess the effects of COVID-19 on cattle traders and the coping mechanism in agro-pastoral and pastoral regions. In this paper, we define a cattle trader as any person engaged in the business of purchasing cattle for the purpose of resale or slaughter (GOU 1943). Although Uganda recorded two lockdowns, our study focuses on the first lockdown largely because we did not have resources to dig deep into the impact of the second lockdown. However, we note that, in the two regions, local governments were allowed to establish local and rural markets in parishes or sub-counties where trucks could come and pick up animals. In the next section, we present the study area, methodology, results, discussion, conclusion, and recommendations.

## Study area

**Fig. 3:**
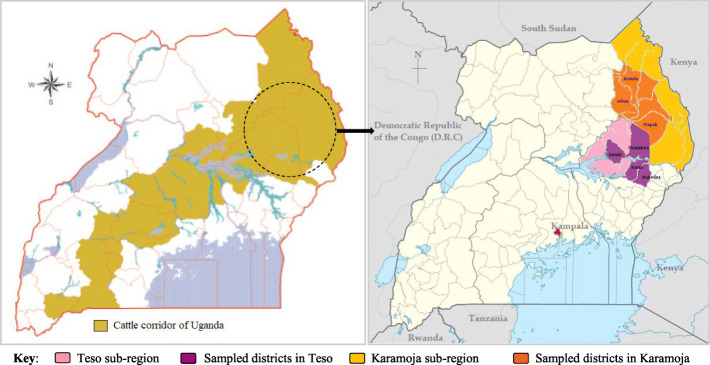
Map of Uganda showing the study area

The study was conducted in Teso and Karamoja sub-regions of eastern and northern parts of Uganda, respectively, as shown in Fig. 3 and have the highest (37%) share of the total cattle population (UBOS 2018a). The Iteso and Karamojong belong to the Nilotic ethnic group, and they are known to have migrated from present-day Ethiopia around 1600 A.D. (Lawrence 1957). The Karamojong are pastoralists with mobile herds, and some are agro-pastoralists while the Iteso were initially agro-pastoralists who have transformed into settled crop farmers combined with cattle keeping (Lawrence 1957; Byaruhanga et al. 2014). The Iteso occupy the districts of Soroti, Kumi, Katakwi, Amuria, Bukedea, Serere, Ngora, Pallisa, Kaberamaido, Tororo, Kapelebyong, and Kalaki, and the Karamojong occupy the districts of Abim, Amudat, Kaabong, Karenga, Kotido, Moroto, Nabilatuk, Nakapiripirit, and Napak. The population in Teso sub-region is estimated at about 2.4 million people with a total land coverage of about 14,855 sq. km (UBOS 2014). While the population in Karamoja sub-region is estimated at about 1.2 million people (pastoralists), with a total land coverage of more than 27,000 sq. km (UIA 2016). In both regions, the most important livestock kept are cattle and goats with sheep being the other key livestock in Karamoja and chicken in Teso.

## Methodology

The study adopted quantitative and descriptive research design with the objective of undertaking both descriptive and inferential statistical analyses to provide a descriptive assessment of the associations between cattle trader characteristics and the likely effects of COVID-19. The target cattle traders were those trading in Teso and Karamoja sub-regional weekly cattle markets. In Teso sub-region, the weekly cattle markets include Ocorimongin market in Katakwi, Kasilo and Ocaapa markets in Serere, Arapai market in Soroti, Odelo market in Kumi, Mukura market in Ngora, and Bukedea market in Bukedea district. In Karamoja sub-region, the main cattle markets include Kanawat market in Kotido, Naitakwae market in Moroto, Komuria livestock market in Kaabong, Lolachat livestock market in Nakapiripirit, and Orwamuge livestock market in Abim district (Loupa 2019).

From the list of the weekly cattle markets above, Bukedea, Odelo, Ocorimongin, and Arapai weekly cattle markets were selected in Teso sub-region representing 57% of the major markets in the sub-region. In the Karamoja sub-region, the Orwamuge, Kangole, and Kanawat weekly cattle markets were selected to be part of the study representing 50% of the major markets in the sub-region. The markets were selected because they are the major busy markets that are easily accessible given the rise in transport costs and restrictions to movement due to COVID-19.

One could argue that markets are not necessarily representative of the sub-region, but we covered 7 out of the 13 major cattle markets which represent 54% of the markets in the region thus representing 54% of the cattle trade in the two sub-regions. Moreover, the selected markets are major busy and accessible markets and handle animals from other markets, and they are the key markets where animals are picked for exports. In addition, the traders are mobile, and they move from market to market, suggesting that these markets handle above-average share of total livestock sales in the two sub-regions. The fieldwork was conducted between February and March 2021 after the lockdown on cattle markets was lifted on October 8, 2020, and each of the cattle markets was visited on its market day.

Since traders are mobile within and across markets, not known in advance, and are difficult to identify in the market, they had to be traced or identified using network sampling popularly known as snowball sampling. Snowball sampling is the best technique used to access hidden and hard-to-reach populations by using one respondent to identify another (Atkinson 2001; Gabor 2007). The method is economical, efficient, and effective while producing reliable and comparable data (Gabor 2007). However, one of the challenges of the snowball sampling is the nature of similarity between the network of traders which may mean ignoring the isolates—those traders who do not have or are not networked (Atkinson 2001). To bring those that were not likely to be part of the specific network, the enumerators were advised to break the network and begin another network by finding a new trader to interview after every three interviews of a given network in the market.

The data was collected using computer-assisted personal interview (CAPI) technology, and a total number of 120 cattle traders (respondents) were interviewed (60 cattle traders from Karamoja and 60 from Teso sub-region) and 7% were females. The data collected included socio-economic variables like age, education, gender access to credit, and region and district of origin or residence. Traders were also asked if COVID-19 affected their cattle trade businesses, and if yes, traders were asked to specify how COVID-19 affected their business. The traders had to select from the following options: (i) I did not sell any animal during lockdown (failed to sell), (ii) I could sell but numbers of animals sold declined (reduced sales), (iii) the cost of transport increased, (iv) the capital declined or eroded, and (v) others. The traders were also asked to follow up questions on each of the listed options. In addition, traders were asked questions regarding how they are coping with COVID-19 lockdown and adherence to COVID-19 SOPs.

The main research questions were as follows: (i) Are there significant differences between cattle traders in Teso and Karamoja in terms of socio-economic characteristics? (ii) Has COVID-19 lockdown affected cattle trade in Teso and Karamoja differently? (iii) How are the cattle traders affected in the Teso and Karamoja? and (iv) How are the cattle traders in Teso and Karamoja responding to the COVID-19 lockdown? To answer these questions, the two-sample test of proportions for the difference in proportion using group and two-sample *t* test for differences in means as well as a two-sample test of proportions using variables to test the changes in pre-COVID-19 and during COVID-19 using the STATA software and the bar graph were used to compare the things between different groups and to track changes in pre-COVID-19 and during COVID-19.

We also estimate the magnitude of the decline (recovery) in the number of cattle sold between pre-COVID-19 (during lockdown) and during the lockdown (after easing of lockdown) as the difference between the number of cattle sold per month by a trader during lockdown (after easing of lockdown) and the number of animals sold pre-COVID-19 (during lockdown). In addition, we also estimated the rate of decline (recovery) by dividing the magnitude of the decline (recovery) by the number of cattle sold between pre-COVID-19 (during lockdown) and multiplied by 100. We then estimated a simple linear regression model to assess the correlates of the magnitude of the decline in the number of cattle sold between pre-COVID-19 and during the lockdown (although we estimated the recovery model, we do not report it because the correlates were not significant). The model can be represented as: ${Y}_i^{\ast }={X}_i{\beta}_i+{\varepsilon}_i$ where ${Y}_i^{\ast }$ is the magnitude of the decline in the number of cattle sold between pre-COVID-19 and during the lockdown, *X*_*i*_ is the asset of independent variables (education level, age, gender, employment level, and cattle trade-related characteristics like transport means), *β*_*i*_ is the unknown parameters, and *ε*_*i*_ is the error term. The variables used are described in Table 1.

**Table 1:** Description of the variables

Variable	Number	Mean	Std. Dev.
Magnititude of the reduction in the number of cattle sold (absolute)	120	3.7	2.5
If the cattle trade treks = 1 and zero otherwise	120	0.4	0.5
If the occupation of the cattle trader was crop farmer = 1 and zero otherwise	120	0.8	0.4
If the cattle trader is from Karamoja = 1 and zero otherwise	120	0.5	0.5
If the cattle trade received COVID-19 support and zero otherwise	120	0.4	0.5
If the cattle trade is male = 1 and zero otherwise	120	0.9	0.2
If the cattle trader completed or attended primary school = 1 and zero otherwise	120	0.7	0.5
If the cattle trader is married = 1 and zero otherwise	120	0.9	0.3
Age of the cattle trader	120	40.5	6.8
If the trade has telephone number = 1 and zero otherwise	119	0.2	0.4
If the trade acquired credit = 1 and zero otherwise	120	0.4	0.5

## Results

### Characteristics of cattle traders by sub-regions 

The results from the *t*-test reveal that there is no significant difference in traders in Teso and Karamoja sub-regions in terms of gender, education, experience in cattle trade, and age (Table 2). However, there is a significant difference between traders in Teso and Karamoja sub-regions in terms of means of transporting livestock, access to credit, means of accessing livestock information, and COVID-19 impacts at a 99% confidence level (Table 2). In Teso sub-region, most cattle traders are more likely to use trucks than to trek compared to the cattle traders from Karamoja, which could be attributed to the fact that Karamoja traders are pastoralists and are used to moving from place to place compared to Teso communities. Also, the most common source of cattle market information was from cattle traders (94%), phones (22%), and radios (16%). For traders in Karamoja, the only source of market information was through cattle traders, while in Teso, the most common source of cattle/livestock market information is through cattle traders (88%), telephones (43%), and radios (32%).

**Table 2:** Characteristics of the cattle traders by sub-region

Variables	Overall	Teso	Karamoja	Difference
Gender of cattle trader (%)				
Male	94	93	95	− 2
Female	7	7	5	2
Education of cattle trader				
Primary	66	65	67	− 2
Secondary	30	33	28	5
Experience in cattle trade	99	103	94	9
Age of cattle trader	41	41	40	1
A cattle trader’s means of transport (%)				
Trekking	43	25	62	− 37***
Trucking	17	32	2	30***
Trekking trucking	48	43	37	6
Access to credit (%)	63	93	32	61***
Credit receipt (%)	42	70	15	55***
Means of accessing market information (%)				
Cattle trader	94	88	100	− 12**
Telephones	22	43	0	43***
Radios	15	32	0	32***
COVID 19 impact (%)	97	93	100	7*

Source: primary data

*p < 0.05, **p < 0.01, ***p < 0.001

The results also reveal that the cattle traders from Teso are more likely to access credit compared to the cattle traders from Karamoja. The main sources of credit for the cattle traders in both Teso and Karamoja sub-regions were village savings, lending associations (VSLAs)^1^ (58.8%), banks (35.2%), and lastly from relatives/friends (5.9%). The cattle traders in the Karamoja sub-region received on average UGX 810,000 (225USD) with a minimum of UGX 50,000 (14USD) and a maximum of UGX 2,000,000 (556USD) while traders in Teso sub-region, farmers received on average UGX 680,000 (189USD) with a minimum of UGX 100,000 (28USD) and a maximum of UGX 3,000,000 (833USD) in Teso sub-region. The main reason for getting credit was as capital to boost the business (60.8%) across Teso and Karamoja sub-regions. Out of the 51 cattle traders who accessed credit, only five were females. All five females accessed credit, and all the 51 traders accessed the credit from the VSLAs.

### Effect of COVID-19 on cattle traders

#### Ways in which COVID-19 affected the cattle traders

**Fig. 4:**
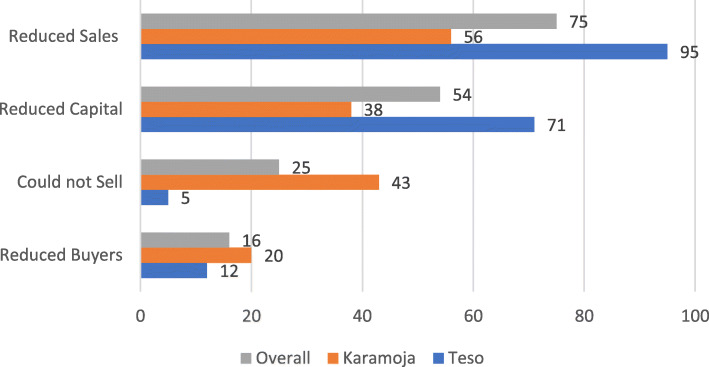
Share (%) of cattle traders reporting ways in which COVID-19 affected cattle business in Teso and Karamoja

Ninety-seven per cent of the cattle traders reported COVID-19 to have affected their cattle businesses (Table 1). The results from the *t*-test reveal that the share of traders affected by COVID-19 was significantly higher in Karamoja compared to Teso sub-region. The cattle traders were also asked to state how COVID-19 affected them. For all the traders interviewed, the ways COVID-19 affected the cattle traders in the order of their importance was reduced sales (75%), reduced or erosion of capital for business (54%), failure to sell animal (25%), and reduced buyers (16%) that drove the prices down (Fig. 4). The results also revealed that the inability to sell animals during COVID-19 was significantly higher among Karamoja traders than Teso traders (*p* value = 0.0000) while capital erosion and reduction in sales were significantly higher in Teso than in Karamoja (*p* value = 0.0003).

### COVID-19 impacts on cattle sales in Teso and Karamoja sub-regions

**Fig. 5:**
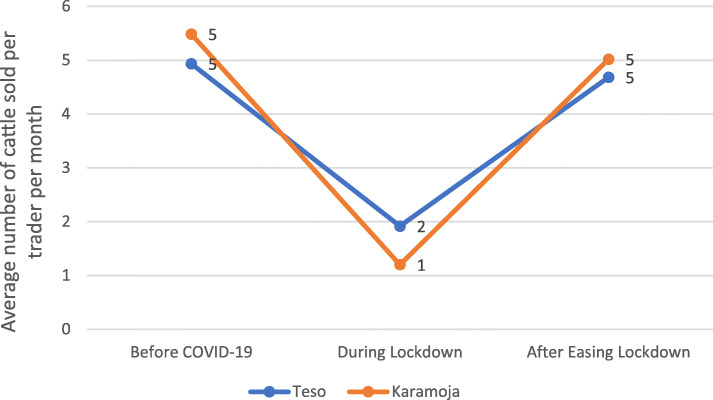
Impact of COVID 19 on average cattle sales in Teso and Karamoja. Source: primary data

The results from this study revealed that the number of animals sold by each individual trader decreased during the lockdown and increased after the easing of the lockdown (Fig. 5). The magnitude of decrease (decline) and increase (recovery) in sales varied by sub-regions. The results from the *t*-test revealed that the reduction in the number of cattle sales was significantly higher in Karamoja compared to Teso sub-region (Table 3). The average number of cattle sold per trade per month declined by 76% in Karamoja and 61% in Teso region.

**Table 3:** Differences in the magnitude of the decline in the number of cattle sold before and during the COVID-19 lockdown by region

Teso (no.)	Karamoja (no.)	Difference	*p* value, mean difference
3.02	4.28	− 1.26	0.002

However, after the re-opening of livestock markets, traders in both sub-regions were able to recover in terms of cattle sales to the pre-closure of livestock markets due to COVID-19 (Fig. 5). Nonetheless, the number of cattle sold by a trader per month was higher in Karamoja compared to Teso before and after COVID-19 restrictions (Fig. 5).

Even though cattle traders in Karamoja had lower cattle sales than those in Teso sub-region during the lockdown, they were able to gain/replenish sales more rapidly than their Teso counterparts after the easing of the lockdown. Karamoja cattle traders registered a significant or greater recovery compared to their Teso counterparts (Table 4).

**Table 4:** Differences in the magnitude of the increase in the number of cattle sold during and after the easing of the lockdown by region

Teso (no.)	Karamoja (no.)	Difference	*p* value, mean difference
2.7	3.8	1.05	0.004

The results from the regression analysis revealed that correlates of the magnitude of the decline in the number of cattle sold per trader per month are means of transporting animals, being crop farmer, and sub-region. The cattle traders that moved on foot (trekked) and those that did not have any employment recorded significantly lower magnitude of the decline in the number of cattle sold between pre-COVID-19 and during the lockdown (Table 5). The results suggest that traders who trek and were initially crop farmer or not involved in any business recorded a low absolute decline in the number of cattle sold per trader per month while those using trucks and had alternative options of employment recorded a higher significant decline in the number of cattle sold per trader per month.

**Table 5:** Correlates of the magnitude of the decline in the number of cattle sold per trader per month

	Model I	Model II
Means of transport is trekking	− 0.981* (− 2.07)	− 0.953* (− 2.11)
A traders’ occupation is being a crop farmer	− 1.323* (− 2.29)	− 1.439** (− 2.79)
A trader is from Karamoja region	1.776** (2.89)	1.856*** (4.12)
A trader is male	0.691 (0.72)	
Attended or completed primary	− 0.339 (− 0.69)	
Being married	− 0.368 (− 0.39)	
Age of the trader	0.0167 (0.46)	
Phone ownership	− 0.100 (− 0.15)	
Acquired credit	− 0.0722 (− 0.13)	
Constant	3.530* (2.00)	4.262*** (9.08)
*N*	119	120

*t* statistics in parentheses

*p < 0.05, **p < 0.01, ***p < 0.001

### COVID-19 impact on expenditure of cattle traders in the different sub-regions

**Fig. 6:**
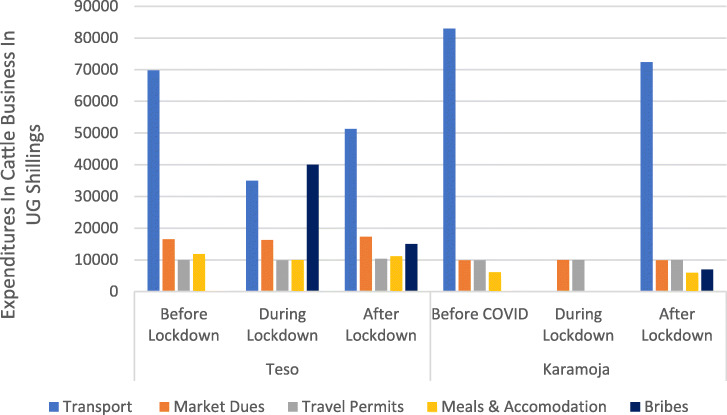
Effects of COVID-19 on the expenditure of the cattle traders. Source: primary data

The results reveal that the main costs that cattle traders incur are transport, market dues, travel permits, meals, and accommodation (Fig. 6). However, due to COVID-19, cattle traders were required or had to pay a fee to the police manning the roadblocks to enforce the lockdown to facilitate movement of the animals (a bribe). In Teso sub-region, the cost emerged during the lockdown and was as high as UGX 40000 (USD 2) but declined after the lockdown to UGX 15,000 (USD 4). In the Karamoja sub-region, the cost appeared only after the lockdown, and it was about UGX 7000 (USD 2). Restriction of movements means that cattle traders must pay a fee to be able to move animals during the lockdown, and this cost was significantly higher in Teso (*p* value = 0.036).

The transport costs were significantly higher before the lockdown compared to during the lockdown (*p* value = 0.0001)*.* Also, the Karamoja traders did not incur any transportation costs during the lockdown but increased sharply after the lifting of the lockdown. After lifting/easing of the lockdown, cattle traders from Karamoja sub-region pay on average UGX 70,000 (USD 20) compared to UGX 50,000 (USD 15) paid by their counterparts in Teso sub-region (*p* value = 0.0000). The costs of market dues, travel permits, meals, and accommodations for cattle traders were not significantly different before, during, and after the lockdown. However, market dues and accommodation costs incurred by Teso cattle traders were slightly higher compared to what the Karamoja traders incur or pay. Meanwhile, the cattle traders from both sub-regions pay the same amount of money, on average UGX 10,000 for the travel permit.

### COVID-19 impact on availability of the cattle buyers and destination outside region

**Fig. 7:**
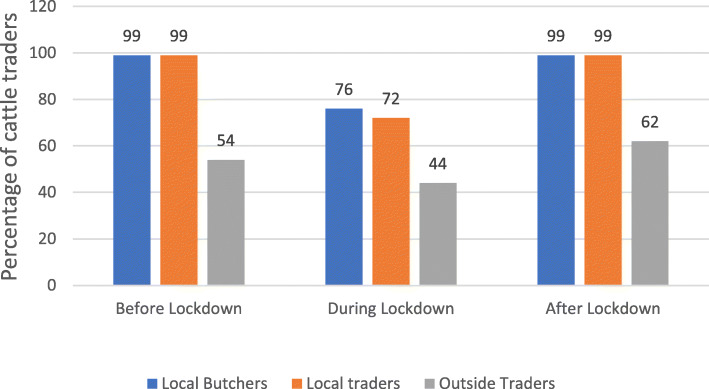
COVID 19 impacts on cattle traders’ interactions with different buyers. Source: primary data

The results show that before COVID-19, the main buyers for cattle from cattle traders from these sub-regions are local butchers, local traders, and traders from outside the region (Fig. 7). However, during the COVID-19 lockdown, trade in the three categories reduced significantly. The most affected was the trade between study participants and local traders (27% reduction), followed by the trade between study participants and local butchers (23% reduction) and the least was the reduction in the trade with traders from outside the trader’s sub-region (10% reduction as shown in Fig. 7). After the easing of the lockdown, the traders in the three categorises normalised to before the COVID-19 period.

**Fig. 8:**
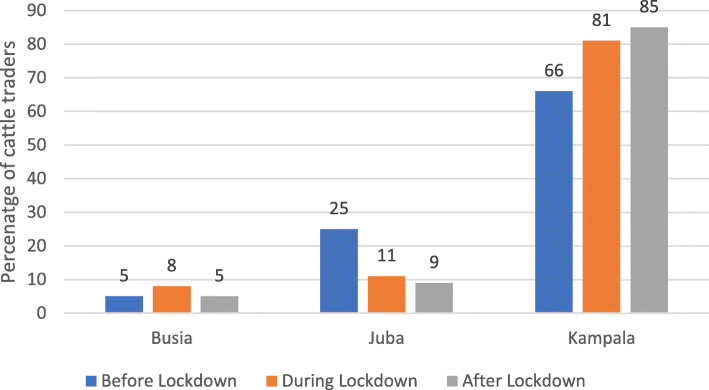
Cattle destination during the different periods for Teso sub-region. Source: primary data

The cattle traders were also asked to specify which traders outside the region they trade with. The main traders they identified are those from Kampala, Busia, and Juba (Fig. 8). The leading destination for cattle from Teso and Karamoja sub-regions during the different periods was Kampala, followed by Juba and Busia Kenya. During the lockdown, the share of cattle traders trading with Busia Kenya traders was slightly higher compared to before and after the lockdown. In the case of Juba, before the lockdown, the share of cattle traders trading with its traders was significantly higher compared to during and after the lockdown (*p* value = 0.005). In fact, the share of cattle traders trading with South Sudan (Juba) even after the lockdown declined. Interestingly, the share of cattle traders dealing directly with traders from Kampala has kept increasing from before, during, and after lockdown (Fig. 8).

### Coping strategies and adoption of standard operating procedures (SOPs)

**Fig. 9:**
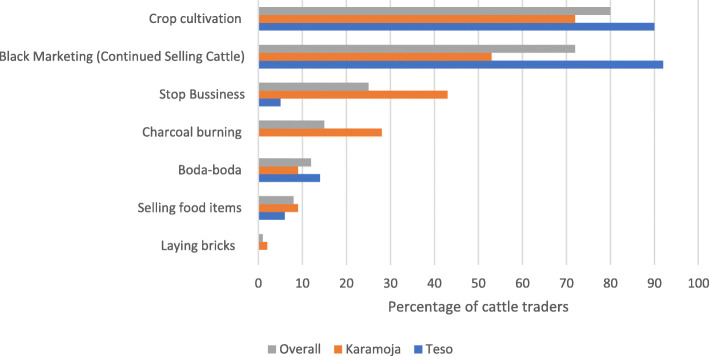
Coping strategies during the closure of the cattle markets. Source: primary data

Cattle traders were asked if they were engaged in any economic activity when the cattle markets were closed due to COVID-19, and 85% replied yes. They were then asked which economic activities they were engaged in, and the results reveal that they engaged in crop cultivation (80%), charcoal burning (15%), boda-boda^2^ riding (12%), selling food items (8%), and continued selling cattle during the lockdown (72%). Most of the cattle traders in Teso (90%) and Karamoja (72%) sub-regions diversified into crop cultivation while charcoal burning and bricklaying was undertaken only in Karamoja sub-region (Fig. 9).

**Fig. 10:**
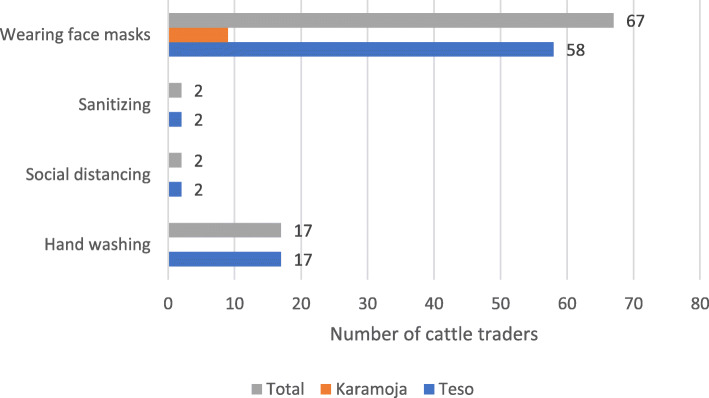
Number of cattle traders practising the COVID-19 SOPs after lockdown. Source: primary data

Cattle traders were also asked if there were SOPs being implemented and followed by traders during the COVID-19 lockdown, and 58% reported that they were. In the Teso sub-region, all the cattle traders reported there were SOPs being implemented and followed by traders during the COVID-19 lockdown while in Karamoja, only 15% reported there were SOPs being implemented and followed by traders during the COVID-19 lockdown thus suggesting low adoption of SOPs in the Karamoja sub-region. Implemented SOPs included hand washing, sanitising, social distancing, and wearing of face masks. Traders in Teso (58 out of 60) were putting on face masks compared to Karamoja (9 out of 60) (Fig. 10). Social distancing, sanitising, and hand washing were least adopted. No trader in Karamoja engaged in social distancing, sanitising, and hand washing. The likelihood of putting on masks was significantly correlated with receiving masks from the government.

**Fig. 11:**
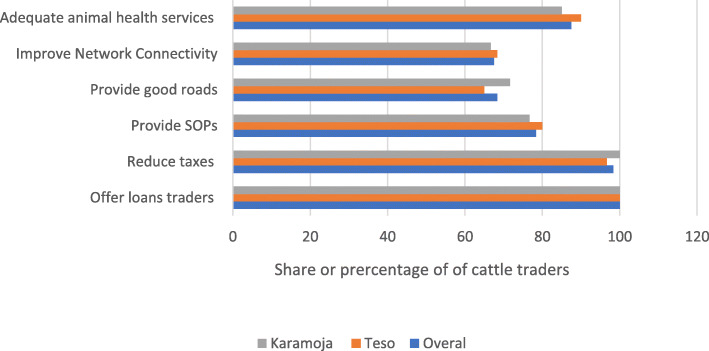
Percentage of cattle traders reporting the key intervention that government could undertake to support cattle trade in the COVID-19 period. Source: primary data

The cattle traders were also asked to state the key intervention that government could undertake to support cattle business during the COVID-19 era. All the traders reported access to loans as the key intervention followed by reduced taxes, provision of adequate animal health services, support with the adoption of SOPs, and improvement in the communication network and roads in the order of importance (Fig. 11).

## Discussion

The COVID-19 pandemic imposed a great threat to the cattle business resulting from the total market closure from April to September 2020. The research findings show that cattle traders were negatively impacted by COVID-19. The main effects of COVID19 on the cattle traders were reduced sales, reduced or erosion of capital for business, failure to sell animals, and reduced number of buyers. The average number of cattle sold per trader per month declined, which is consistent with the previous study that also found a decrease in cattle sales at the beginning of the pandemic (Lynch 2020). Karamoja traders’ cattle sales declined to a greater extent compared to Teso traders. This can largely be attributed to the fact that the cattle markets in Karamoja are very remote and insecure, and buyers are unable to access these markets during the lockdown. In addition, the higher costs of fuel, costs of vehicle service, and costs of acquiring spare parts are very high because of poor roads and result in many traders preferring to trek or move on foot to save costs. However, trekking was associated with a greater decline in sales because of COVID-19 restrictions on human movement.

The reduction in sales, erosion of capital for business, failure to sell animals, and reduced number of buyers are a threat to food security (Griffith et al. 2021). In pastoral areas, trade in livestock is key to pastoral livelihoods as it provides income that can be used to purchase non-animal source foods, household basic needs, and inputs for livestock production (Little et al. 2014). The cattle trader’s inability or failure to sell animals limits their ability to purchase animals from the pastoralists who have animals to sell (KRSU, 2018). These leave both traders and pastoralists with no income to buy food, other basic needs, and animal health care services, thus leading to increased livestock mortality (Griffith et al. 2021). Also, consumers are left vulnerable to malnutrition because of the reduced supply of animal source foods which are the main source of high-quality proteins and micronutrients (Griffith et al. 2021; Mitscherlich et al. 2021).

Following the lifting of the lockdown, traders in Karamoja were more likely to re-engage in cattle trading compared to the Teso traders. This can be attributed to the fact that they did not engage in alternative livelihoods to the same extent. In Teso, traders engaged in crop cultivation, retail business, and boda-boda riding to a great extent. These livelihood diversification strategies have been shown to be less affected or impacted by COVID-19 (Aziz et al. 2021). However, traders in Teso who pursued this strategy did report more difficulty in re-engaging in cattle trade once the lockdown was lifted. Karamojong traders could not easily diversify because they have a low asset base, are dependent mainly on livestock, and crop farming is not their main livelihood (Achiba 2018; McCabe et al. 2010; McCabe et al. 2014).

Also, most of the traders who reported to have lost capital were mainly in Teso sub-region. This could be attributed to fact that they had to pay higher costs of transaction to facilitate trade during the lockdown. In addition, some had to spend money to pay school fees which eroded their capital. As shown in the results, the cost incurred by Karamoja traders during the lockdown was very low, and when the market was closed, they also closed the business. In addition, parents in Karamoja generally do not send children to school and therefore do not have many obligations to pay fees as most of the children are raised to graze cattle (Agiresaasi and Nakisanze 2019). Indeed, the gross and net enrollment rates are lowest in Karamoja (36%) compared to Teso (87%) and other regions in Uganda (UBOS 2018b).

The results also show that the traders in both sub-regions have failed to recover the international markets like South Sudan and Kenya after the lockdown. The share of traders trading with Juba and Busia is declining while the share trading with Kampala is increasing. These results are consistent with many studies that have shown that COVID-19 has led to a decline in trade in goods and services, especially those that require human movement like cattle traders who need to move with their animals to ensure that they are not cheated, and the animals safe (Minondo 2021). Moreover, organised cattle thefts or raids intensified during the COVID-19 lockdown and have led to the loss of livestock and consequently a reduction in livestock production and sales. In addition, the concentration of the security personnel on COVID-19 response activities limited informal cross-border cattle trade by locking all borders (IPC 2021).

Although the new trend of the establishment of new livestock markets driven largely by the formation of new sub-counties and with the objective of raising revenues for the local government has been observed (KRSU 2018), it seems that COVID-19 lockdown and closure of livestock markets affected this trend. The decline in the regional and international trade drove cattle traders to develop networks within the country to enable them to do business, thus explaining why the share of traders trading with Kampala traders is on the rise. However, this can only be sustainable if the beef or meat industry or sector is able to establish cold chains and traceability systems to enable to access international markets and reduce disruption or stabilise the beef/cattle markets (Trotter and Michael 2020). This should be backed up with a strong enforcement of the food safety regulations and standards (Aday and Aday 2020; Ijaz et al. 2021). Even if the restrictions are removed, the future of the meat industry requires these facilities and systems to be in place to promote trade in meat products. Already, the government is investing and continues to advocate for investments in these facilities and systems under the National Development Plan III (NPA 2020).

To cope up with the closure of the cattle markets, most of the cattle traders had to diversify to other alternative livelihoods and significant numbers, especially those from the Teso sub-region stayed in business selling cattle but this time not at the markets but through phones since they have better phone coverage than their Karamojong counterparts (UBOS 2018a). Since the movement of agricultural produce was allowed under the lockdown conditions, traders would receive the orders by phone and be able to deliver. Traders in Karamoja, a majority of which do not have phones (UBOS 2018b), were disadvantaged and therefore could not stay in business.

Among the standard operating procedures (SOPs) implemented by cattle traders, the results show that most of them in Teso sub-region observed hand washing and wearing of face masks during the market re-opening as compared to traders from Karamoja. In Karamoja, the masks were scarce and expensive for the community (Lotira et al. 2020), and putting on masks was correlated with receiving the mask from the government and yet the distribution of masks in Karamoja was generally difficult (Ebele 2020). The weakness in the distributions of the masks could be associated with weakness in the District COVID-19 Task Force. Indeed, the results of the assessments of the performance of the COVID-19 District Task Force revealed that Karamojong communities represent the highest proportion of community members reporting to have missed out on food distribution completely at 91% (Mbabazi and Kasalirwe 2020).

## Conclusion and recommendation

COVID-19 has negatively affected cattle traders in many ways including reduced sales, reduced or erosion of capital for business, failure to sell animals, and reduced number of buyers thus limiting the ability of the pastoralists to sell their animals and worsening food security because income from livestock is used to buy non-animal source food. To support cattle traders to fully recover and continue in the business and ensure the resilience of the pastoral livelihood, cattle traders will need to be supported with loans or credit to boost their reduced capital and enable them to continue operating the newly discovered economic activities as risk mitigation strategy while also engaging themselves in the cattle trade. This can be achieved by capitalising VSLAs where these traders belong. Moreover, in pastoral areas access to communication and good road network should be boosted to enable these traders to be able to participate in the cattle trade.

In addition, the declining regional and international trade means that there is a need to support the meat industry or sector to establish standard slaughter facilities with cold chains to reduce cattle movements but rather trade in quality beef or meat. This should be supported with a good traceability system as well as strong and effective food quality and safety regulations and rules to enable to access international markets and reduce disruption or stabilise the beef/cattle markets while securing pastoral livelihoods. Lastly, the cattle traders should be provided with masks and promote the adoption of SOPs to protect them from the COVID-19 pandemic and reduce the need for lockdown and cattle market closures which are detrimental to pastoral livelihoods.

## Data Availability

Data are available on reasonable request.

## Abbreviations

CAPI: Computer-assisted personal interview
COVID: Coronavirus disease
DANRE: Department of Agribusiness and Natural Resource Economics
IRB: Institutional Review Board
KSU: Kansas State University
SOPs: Standard operating procedures
UBOS: Uganda Bureau of Statistics
UIA: Uganda Investment Authority
UN: United Nations
UNICEF: United Nations International Children’s Fund
VSLA: Village Savings Lending Association
NPA: National Planning Authority
NDP III: Third National Development Plan

## Footnotes

1 A Village Savings and Loan Association (VSLA) is a group of people who meet regularly to save together and take small loans from those savings. The activities of the group run in cycles of one year, after which the accumulated savings and the loan profits are distributed back to the members. The purpose of a VSLA is to provide simple savings and loan facilities in a community that does not have easy access to formal financial services.

2 Boda-Boda (motorcycle taxis) riding – is one of the popular means of public transport in Uganda and accounts for a significant proportion of all motorized trips in the country. Their popularity, often attributed to their maneuverability within traffic and their easy navigation of untarred terrain. Riding is the activity of riding or cycling a motor bike or bicycle.
